# Thoracoscopic Resection of an Esophageal Schwannoma Demonstrating a Malignant Pattern on Dual-Time-Point FDG-PET: A Case Report

**DOI:** 10.70352/scrj.cr.25-0179

**Published:** 2025-06-24

**Authors:** Takuya Harada, Konomi Takemoto, Naoki Okada, Nozomi Minagawa, Yoshihiro Matsuno, Yoshiaki Maeda

**Affiliations:** 1 Department of Gastroenterological Surgery, Hokkaido Cancer Center, Sapporo, Hokkaido, Japan; 2 Department of Pathology, Hokkaido Cancer Center, Sapporo, Hokkaido, Japan

**Keywords:** esophageal schwannoma, thoracoscopic surgery, dual-time-point FDG-PET, minimally invasive surgery

## Abstract

**INTRODUCTION:**

Esophageal schwannomas are rare benign tumors arising from Schwann cells and are among the least common mesenchymal neoplasms of the gastrointestinal tract. While fluorodeoxyglucose positron emission tomography (FDG-PET) is widely used in the preoperative assessment of submucosal tumors, schwannomas are known to exhibit unexpectedly high FDG uptake, often mimicking malignant lesions. Dual-time-point FDG-PET, which evaluates both early and delayed FDG accumulation, has been employed to improve diagnostic specificity in various malignancies, including esophageal cancer. However, to date, no cases of esophageal schwannomas showing a malignant FDG uptake pattern on dual-time-point FDG-PET and resected thoracoscopically have been reported. We present a rare case of esophageal schwannoma demonstrating increased delayed FDG uptake, initially suggestive of malignancy that was successfully treated with thoracoscopic surgery following preoperative diagnosis via EUS-FNA.

**CASE PRESENTATION:**

A 55-year-old woman was referred to our hospital for evaluation of an esophageal submucosal tumor detected during health screening. FDG-PET demonstrated a high uptake pattern (SUVmax; early; 9.4, delayed; 11.8) suggestive of malignancy. However, endoscopic ultrasound-guided fine-needle aspiration (EUS-FNA) revealed histopathological features consistent with a benign schwannoma. The patient underwent thoracoscopic enucleation, and the intraoperative findings confirmed a well-circumscribed tumor without invasion of the surrounding tissues. The procedure was completed within 2 h and 5 min with minimal blood loss. The final pathological diagnosis confirmed esophageal schwannoma. The postoperative course was uneventful, and the patient was discharged on postoperative day 14. At the 6-month follow-up, no recurrence was observed.

**CONCLUSIONS:**

This case highlights that even when dual-time-point FDG-PET suggests malignancy, thoracoscopic resection may be a viable treatment option for esophageal schwannoma if a benign diagnosis is supported by preoperative EUS-FNA. This underscores the importance of integrating metabolic imaging with histopathological assessment in surgical planning.

## Abbreviations

ADC
: apparent diffusion coefficient
DOG-1
: discovered on GIST-1
DWI
: diffusion-weighted imaging
EUS
: endoscopic ultrasound
EUS-FNA
: endoscopic ultrasound-guided fine-needle aspiration
FDG-PET
: fluorodeoxyglucose positron emission tomography
Ki-67
: proliferation marker Ki-67 antigen
SMA
: smooth muscle actin
SOX10
: SRY-related HMG-box 10
SUVmax
: maximum standardized uptake value

## INTRODUCTION

Schwannomas are rare benign tumors originating from the Schwann cells of the nerve sheath and are exceptionally uncommon.^1,2)^ Initial diagnostic evaluations typically involve CT, MRI, PET, and endoscopic examinations.^3,4)^ EUS-FNA may facilitate pathological diagnosis. Among these, FDG-PET is a valuable tool for preoperative planning; however, schwannomas often exhibit high FDG uptake, which may mimic malignant tumors.^5,6)^ Numerous studies have demonstrated a temporal correlation between standardized uptake value (SUV) and FDG accumulation, with tumor tissues exhibiting a progressive increase in FDG uptake for several hours following FDG administration. By contrast, such sustained uptake is uncommon in inflammatory, infectious, or normal tissues.^7,8)^ Dual-time-point FDG-PET, which utilizes both early and delayed FDG uptake measurements, has been applied to the diagnosis of various malignancies,^9,10)^ and several studies have reported its utility in the evaluation of esophageal cancer.^11)^

The primary treatment for schwannomas is surgical resection, with minimally invasive approaches, such as thoracoscopic or laparoscopic surgery, being increasingly used because of their favorable outcomes.^2,12)^ Malignant transformation of schwannomas is extremely rare,^13)^ and complete surgical resection generally leads to favorable prognosis, with most patients achieving long-term disease-free survival.^1,2)^

To our knowledge, there have been no previous reports of thoracoscopic resection of an esophageal schwannoma exhibiting a malignant pattern on dual-time-point FDG-PET—specifically, with delayed FDG uptake exceeding early uptake. Herein, we describe such a rare case encountered at our institution.

## CASE PRESENTATION

A 55-year-old woman was referred to a local hospital after an abnormal shadow was detected on chest radiography during routine health screening. Subsequent computed tomography at that institution revealed a mediastinal mass, and PET-CT demonstrated high FDG uptake. She was then referred to our hospital for further evaluation and management. The patient had no specific symptoms and physical examination was unremarkable.

**Fig. 1:**
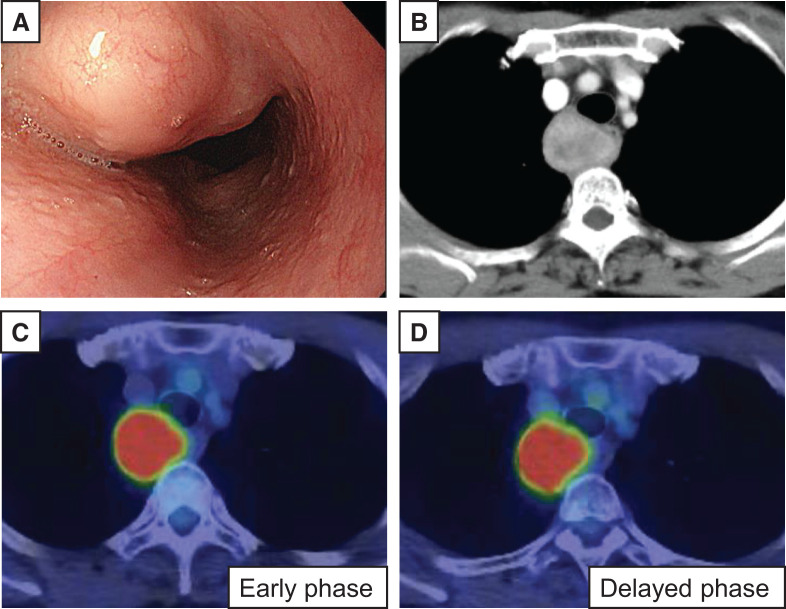
(**A**) Upper gastrointestinal endoscopy revealed a well-demarcated, smooth-surfaced lesion in the upper esophagus, 17 cm from the incisors, without luminal obstruction. (**B**) Chest CT identified a 34 × 27 mm mass on the right side of the upper thoracic esophagus, exerting compression on both the trachea and esophagus. (**C**, **D**) FDG-PET revealed elevated uptake, with an early SUVmax of 9.4 (**C**) and a delayed SUVmax of 11.8 (**D**). FDG-PET, fluorodeoxyglucose positron emission tomography; SUVmax, maximum standardized uptake value

Upper gastrointestinal endoscopy revealed a smooth-surfaced elevated lesion located in the upper esophagus, 17 cm from the incisors, which did not obstruct the passage of the scope (**Fig. 1A**). EUS with radial scanning revealed a 38 mm well-defined, round, hypoechoic mass located within the submucosal layer of the esophageal wall and extending into the posterior mediastinum. Bronchoscopy revealed an external compression of the lower membranous trachea without mucosal involvement.

Chest CT revealed a 34 × 27 mm tumor located on the right side of the upper thoracic esophagus, compressing the trachea and esophagus (**Fig. 1B**). MRI confirmed the presence of a tumor, which appeared hypointense on T1-weighted imaging, mildly heterogeneous and hyperintense on T2-weighted imaging, and hyperintense on diffusion-weighted imaging without an apparent reduction in apparent diffusion coefficient values. FDG-PET demonstrated increased uptake, with an early SUVmax of 9.4 (**Fig. 1C**) and a delayed SUVmax of 11.8 (**Fig. 1D**), raising the possibility of malignancy.

Histopathological examination of specimens obtained via EUS-guided biopsy revealed SOX10 positivity and negativity for CD34, SMA, and desmin, with an extremely low Ki-67 labeling index, suggestive of a schwannoma. Therefore, the preoperative diagnosis was benign esophageal schwannoma.

**Fig. 2:**
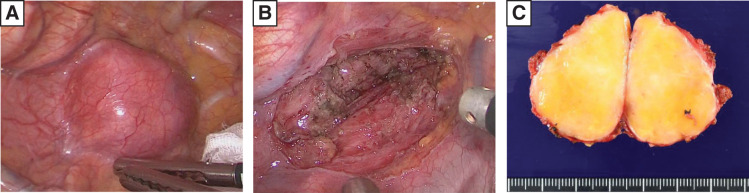
(**A**) Intraoperative thoracoscopic imaging revealed a smooth-surfaced tumor at the upper mediastinum. (**B**) Although the tumor exhibited broad adhesion to the esophageal muscular layer, careful dissection and removal of the membranous structures containing fine nerves enabled complete resection without damaging the esophageal musculature. (**C**) Macroscopically, the tumor was a well-encapsulated, solid mass measuring 40 × 40 × 30 mm, with a yellowish-white appearance.

The patient underwent thoracoscopic tumor resection (**Fig. 2A**). The patient was placed in the prone position and trocars were placed in the 3rd, 5th, 7th, and 9th intercostal places. Intraoperatively, the tumor was easily dissected from the trachea due to the presence of loose connective tissue between the 2 structures. Although the tumor broadly adhered to the esophageal muscular layer, careful dissection and removal of the intervening membranous structures containing fine nerves allowed complete excision of the tumor. While partial resection of the muscular layer was necessary, it was limited in extent, and the mucosal surface remained intact without exposure (**Fig. 2B**). The procedure lasted for 2 h and 5 min, with a total blood loss of 4 mL.

**Fig. 3:**
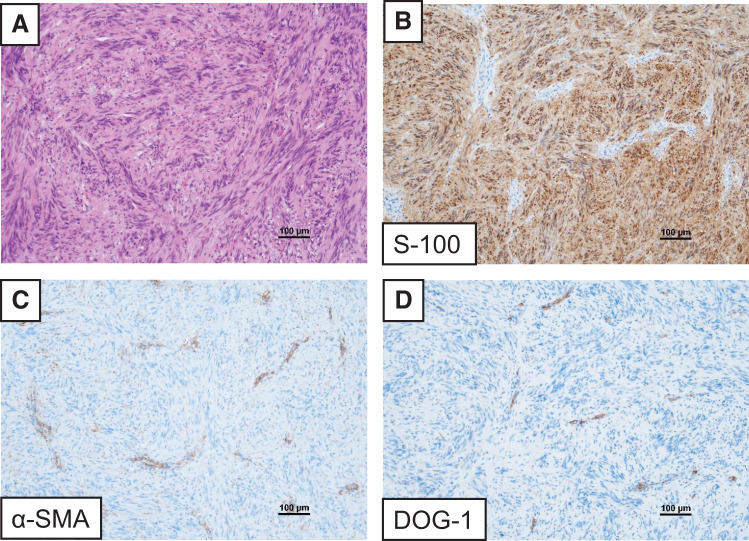
(**A**) Histological examination revealed a spindle cell tumor arranged in a loose fascicular or whorled pattern, with no signs of malignancy. (**B**) Immunohistochemical staining demonstrated S-100 positivity, (**C**) while α-SMA and (**D**) DOG-1 were negative. DOG-1, discovered on GIST-1; SMA, smooth muscle actin

Postoperative pathological examination revealed a well-encapsulated, solid, yellowish-white tumor measuring 40 × 40 × 30 mm with a fibrous capsule (**Fig. 2C**). Histologically, the tumor comprised interwoven spindle cells arranged in a loose fascicular or whorled pattern without evidence of malignancy (**Fig. 3A**). Immunohistochemical analysis demonstrated positive staining for S-100 (**Fig. 3B**) and negative staining for α-SMA (**Fig. 3C**) and DOG-1 (**Fig. 3D**), confirming the diagnosis of a benign esophageal schwannoma.

The postoperative course was uneventful. The patient resumed oral intake on postoperative day 3 and was discharged without complications on postoperative day 14. 6 months postoperatively, the patient remained recurrence-free.

## DISCUSSION

Esophageal schwannomas are extremely rare mesenchymal tumors, first described by Chaterlin and Fissore, in 1967, and remain the least common among gastrointestinal schwannomas.^14)^ Due to their nonspecific clinical presentation and submucosal growth pattern, preoperative diagnosis is often challenging.^3)^ While cross-sectional imaging modalities such as CT and PET-CT provide information on tumor size and localization, they are frequently insufficient to establish a definitive diagnosis.^4)^

FDG-PET, although widely used in oncologic imaging, is particularly problematic in this context. Increased FDG uptake is typically interpreted as a hallmark of malignancy because of its association with enhanced glucose metabolism in cancer cells. Accordingly, a high SUV is frequently used as an indicator of malignant potential. However, this approach lacks specificity. Benign lesions such as schwannomas, inflammatory masses, and granulomatous disease can also demonstrate elevated SUVmax, resulting in potential false-positive interpretations.

For instance, Miyake reported that benign schwannomas may exhibit SUVmax values exceeding 5.0, overlapping with those seen in malignant peripheral nerve sheath tumors (MPNSTs).^15)^ Similarly, Wang demonstrated that SUV max values in benign schwannomas ranged from 2.4 to 9.2, and emphasized the risk of misdiagnosis when interpreting FDG-PET findings in isolation.^16)^

Several histopathologic and biologic mechanisms have been proposed to explain the hypermetabolic appearance of benign schwannomas. Wang identified peritumoral lymphoid cuffs in approximately 80% of schwannomas and found a significant correlation with SUV max, suggesting that peritumoral inflammation may contribute to increased FDG uptake.^16)^ In addition, overexpression of glucose transporters—especially GLUT1 and GLUT3, which are abundant in neural tissue—has also been implicated.^15)^ Anatomical site may further influence FDG accumulation, with tumors located in the gastrointestinal tract and abdominal cavity tending to show higher SUVmax than those in other locations.^16)^

To improve the specificity of PET-based diagnosis, dual-time-point FDG-PET has been proposed. Malignant tumors generally exhibit higher FDG uptake during the delayed phase compared with the early phase, while benign or inflammatory lesions tend to demonstrate stable or reduced uptake over time.^11,17,18)^ In our case, the early SUV max was 9.4 and the delayed SUVmax was 11.8, producing a retention index >10%, a pattern typically associated with malignancy. Xiu demonstrated that using a retention index >10% achieved a diagnostic accuracy of 84.8% for malignancy.^19)^

However, the findings in this case challenge the reliability of dual-time-point FDG-PET for differentiating benign from malignant lesions. A PubMed search using the terms “esophageal schwannoma OR esophageal nerve sheath tumor” AND “thoracoscopic surgery” identified 10 publications, among which 7 studies (10 cases) described thoracoscopic resection of esophageal schwannomas. Only 2 of these reports included any description of FDG-PET findings,^20,21)^ and none reported dual-time-point FDG-PET values or demonstrated the malignant FDG pattern observed in our case (**Table 1**). To the best of our knowledge, this is the first report of a thoracoscopically resected esophageal schwannoma showing a malignant pattern on dual-time-point FDG-PET.

**Table 1:** Reported cases of thoracoscopic resection of esophageal schwannomas

Case	Age	Sex	Tumor size (mm)	SUVmax (FDG-PET)	Dual time FDG	Patology	Author	Year
1	23	M	30 × 20 × 50	N	N	Benign	Schmid	1997
2	46	M	30 × 18 × 15	N	N	Benign	Chen	2016
3	42	F	30 × 40 × 40	N	N	Benign	Chen	2016
4	58	F	80 × 60 × 50	N	N	Benign	Chen	2016
5	39	F	39 × 28 × 56	5.5	N	Benign	Watanabe	2016
6	57	F	38 × 30	N	N	Benign	Hu	2017
7	74	F	35 × 30 × 25	12.3	N	Benign	An	2019
8	52	M	65 × 40 × 45	N	N	Benign	Zhang	2021
9	76	M	80 × 60 × 80	N	N	Benign	Zhang	2021
10	58	M	60 × 52 × 42	N	N	Benign	Nakagawa	2024

SUVmax, maximum standardized uptake value; FDG-PET, fluorodeoxyglucose positron emission tomography

Given the diagnostic ambiguity, pathological confirmation remains essential. In our case, EUS-FNA enabled the preoperative identification of a benign schwannoma, thereby avoiding unnecessary esophagectomy and allowing for curative, minimally invasive resection via thoracoscopic surgery. This reinforces the importance of a multimodal diagnostic strategy integrating anatomical, metabolic, and histological assessments to ensure accurate diagnosis and optimal surgical planning.

## CONCLUSIONS

To date, no prior reports have described the use of dual-time-point FDG-PET in esophageal schwannomas. Although the lesion in our case demonstrated a malignant uptake pattern, the findings suggest that, when supported by a reliable preoperative pathological diagnosis such as that obtained via EUS-FNA, thoracoscopic resection may still be considered as a feasible treatment option.

## DECLARATIONS

### Funding

This research did not receive any specific grant from funding agencies in the public, commercial, or not-for-profit sectors.

### Authors’ contributions

TH wrote the manuscript.

All authors read and approved the final manuscript.

### Availability of data and materials 

No datasets were generated or analyzed during the current study.

### Ethics approval and consent to participate

This work does not require ethical considerations or approval.

Informed consent to participate in this study was obtained from the patient.

### Consent for publication

Informed consent for publication of this case report was obtained from the patient.

### Competing interests

The authors declare that they have no competing interests.
